# Synthetic and Non-synthetic Cannabinoid Drugs and Their Adverse Effects-A Review From Public Health Prospective

**DOI:** 10.3389/fpubh.2018.00162

**Published:** 2018-06-07

**Authors:** Koby Cohen, Aviv M. Weinstein

**Affiliations:** Behavioral Science, Ariel University, Science Park, Ariel, Israel

**Keywords:** cannabis, synthetic cannabinoids, novel psychoactive drugs, drug abuse, addiction

## Abstract

There is a growing use of novel psychoactive substances containing synthetic cannabinoids. Synthetic cannabinoid products have effects similar to those of natural cannabis, yet, these drugs are more potent and dangerous, and have been associated with dangerous adverse effects. Here, we review current literature on the epidemiology, acute, and chronic effects of synthetic and natural cannabinoid-based drugs. Synthetic drugs contain a mixture of psychoactive compounds that mostly bind cannabinoid receptors with high potency. These synthetic drugs replicate the effects of natural cannabis and Δ9-tetrahydrocannabinol but they induce more severe adverse effects including respiratory difficulties, hypertension, tachycardia, chest pain, muscle twitches, acute renal failure, anxiety, agitation, psychosis, suicidal ideation, and cognitive impairment. Chronic use of synthetic cannabinoids has been associated with serious psychiatric and medical conditions and even death. Given the growing popularity in the use of cannabinoid-based drugs and their harmful potential, there is a need for further research in this field.

## Introduction

Cannabis is the most widely available and used drug across the world (1, 2). According to the United Nations Office on Drugs and Crime (UNODC) ~4% of the global adult population have used cannabis in their life. In the United States of America (USA) alone, 11% (36 million people) of adults used cannabis at least once in their past (3). In addition, the therapeutic use of cannabis and its derivatives is increasing and has been evaluated for a various health conditions including; pain, anorexia, side-effects of chemotherapy, multiple sclerosis, and muscle spasms (4–6). The primary psychoactive constituent within cannabis is Δ-9 tetra-hydro-cannabinol (THC), which interacts with CB_1_ and CB_2_ receptors and it consequently elicits its main effects (7–9). Novel Psychoactive Substances (NPS) which contain Synthetic Cannabinoids (SCs) have recently started to be used recreationally, especially by young adults (10, 11). In contrast to the decline in use of many NPSs such as the cathinones and piperazines, it appears that the number of SC users is increasing (12). Although SC drugs mimic the psychotropic effects of cannabis, their undesired effects are unpredictable and more severe than those associated with cannabis (10, 13–16). Although, there is an increasing interesting on the therapeutic potential of cannabinoid-based medications (6, 17) repeated exposure to cannabinoid-agonists in either organic or synthetic forms is associated with both physically and psychological adverse effects (2, 10, 15, 18). The most notorious psychological side effects are mental disorders including psychotic-states, schizophrenia, and affective disorders (1, 2, 10). The aim of the current review is to describe the available knowledge regarding acute and repeated consumption of both organic and synthetic cannabinoid drugs and their side effects from a public health prospective.

## Epidemiology and pattern of use of cannabinoids

According to national and regional representative surveys, lifetime prevalence of SC use in the general population is between 0.2 and 4% (19). By comparison, lifetime use of cannabis tends to be greater; and to range between one-quarter to one-third of the population (1). However, Winstock and Barratt reported that SC products are widely popular among recreational cannabis users (20). Among high school seniors in USA, the annual prevalence of SC usages was higher than any other drugs, with the exception of cannabis (21). Evidence accumulated from several surveys shows that between 6 and 17% of college students in USA have used SC drugs at least once during their study period (22, 23). Other than that, SC use was relatively frequent among adolescents and young-teenagers. Approximately 1% of European people between the ages of 14–18 used SC drugs at least once in their lifetime (24). This is especially important since both clinical and preclinical studies indicate that exposure to SC as well as THC during adolescence is associated with an increased risk of developing schizophrenia later in life (25–27) (see Table 1 for comparison of cannabis and SC adverse effects). Prompted by the alarming growth of the SC drugs phenomenon, legal measures to control the distribution of these drugs have been taken in many countries (57, 58). For example, in the United Kingdom (UK), “first generation” SCs were controlled in 2009 and further legislation to control so-called “second generation” of SCs drugs was enacted in 2013. Yet, subsequent manipulation of the chemical structure of these compounds has resulted in a novel generation of SC's that are not currently legally controlled in the UK (57). Unfortunately, a similar pattern was observed in other countries as well, manufacturers of SCs are aware of the chemical analog loopholes in the law and continue to manipulate SCs as necessary to keep them legal for distribution (26, 57–59). In line with this, a recent epidemiological study by Waugh et al. (57) reported substantial increase in SC drug use in 2011 despite prior legislation efforts. Suggesting, that legislation efforts alone have an insufficient effect on the distribution and use of SC drugs, and further prevention efforts are required to control this phenomenon (57).

**Table 1:** Summary of acute and long-term clinical side-effects of cannabinoid-based drugs.

**Symptoms**	**Type of effect^+^**	**Type of drug**	
		**Synthetic Cannabinoids**	**Cannabis**
Neuropsychiatric	Acute	Severe psychotic symptoms including; agitation (28), aggression, catatonia, paranoia, auditory and visual hallucinations, perceptual alterations, and persistent psychosis episode (10, 14, 29).	Perceptual alterations including; hallucinations and distortion of spatial perception are typical effects (7, 30). Paranoia, aggressiveness, and prolonged psychosis were observed in vulnerable users and are dose-related (1, 2, 7).
	Long-term	Chronic use may increase the risk for developing psychotic disorders (15, 27, 31).	An increased risk of psychotic disorders in vulnerable individuals and naïve users (2, 25, 27, 32).
Affect	Acute	Negative mood, panic attacks, manic behavior (13), depression (16), and suicidal ideation (10, 33).	Anxiety and panic attacks; especially in naïve users (1).
	Long-term	Depression (16), irritability and persistent anxiety (29, 33).	An increased risk for developing anxiety (34, 35), and mood disorders (1, 36, 37).
Cognitive	Acute	Severe cognitive impairments including; memory alteration, attention difficulties, and amnesia (13, 33).	Wide range of dose-related cognitive deficits including; attention, working-memory, cognitive inhibition, and psychomotor function (38–41).
	Long-term	Executive function deficits of working memory and attention (16)	Impairments of set-shifting, verbal learning, attention, short-term memory and psychomotor functions (39, 42).
Cardiovascular	Acute	Tachycardia, hypertension, myocardial infraction, arrhythmias, chest pain, and palpitations (13, 43).	An increase of cardiovascular activity, increase heart rate, and decrease blood pressure (44, 40).
	Long-term	Prolong use may increase risk of cardiovascular disease (44, 45).	An increased risk of cardiovascular disease after prolong use (1, 44, 46).
Neurologic	Acute	Dizziness, somnolence, seizures, hypertonicity, hyperflexion, hyperextension, sensation changes, and fasciculations (10, 14, 29).	Dizziness, somnolence, and muscle tension (38, 40).
	Long-term	Preliminary evidence for structural and functional central nervous system alterations (47, 48).	Structural and functional abnormalities in a range of brain areas including the hippocampus and amygdala (49, 50).
Gastrointestinal	Acute	Nausea, emesis, and appetite change (10, 14, 20, 29).	Hyperemesis, and increase appetite (1, 7, 20).
	Long-term	Severe weight-loss after prolong use (10, 13).	Low body weight among regular users (51).
Other	Acute	Acute kidney injury, abdominal pain, miosis, mydriasis, xerostomia, hyperthermia, fatigue, rhabdomyolysis, cough (11, 13, 43), deficits of driving ability (52, 53, 54).	Bronchodilation (55), impairments of driving ability (1, 7).
	Long-term	Kidney diseases, insomnia, nightmares, dependency, tolerance, and withdrawal (11, 13, 43).	An increased risk of obstructive lung disease including lung-cancer (1, 7, 55), an increased risk of cancers of the oral cavity, pharynx and esophagus (56), cannabis addiction, tolerance, and withdrawal (1, 7).

+ *Acute effect denotes 0–6 h after last drug use; Long-term effects denotes 3 weeks or longer after last drug use*.

Naive consumers typically report using SCs for various reasons, such as curiosity, high availability, easy access, and lower costs compared with cannabis. Since SCs are mostly undetectable via a simple urine test, a major motivation for consuming SC drugs is the desire to experience “cannabis-like” effects without the danger of being detected (11). Other motivations to use SCs are their relatively high availability and low prices (11, 19). In in contrast to cannabis, these synthetic drugs are typically not designed to be mixed with tobacco, probably to achieve the most intense effects (13).

## Psychoactive ingredients of cannabinoids

The main psychoactive ingredient in cannabis is THC, which is a CB_1_ and CB_2_ receptors partial-agonist and the most potent cannabinoid that is present in the organic forms of cannabis (8, 27). Besides THC, organic cannabis products contain additional cannabinoids which do not induce psychoactive effects, such as Cannabinol, Δ8-Tetrahydrocannabinol, and Cannabidiol (CBD) (9, 60, 61). CBD is considered a non-psychoactive cannabinoid that also moderates the psycho tropic effects of THC (32, 62). Moreover, evidence is increasing that CBD has anxiolytic and antipsychotic properties (17, 62). In a broader context, CBD appears to have the ability to counteract psychotic symptoms and cognitive impairment associated with cannabis use as well as with acute THC administration (17, 63–65).

In contrast to cannabis, which contains mostly a mixture of agonist and antagonist cannabinoids (1, 7). SC's compounds show differences in their selectivity, their potency and their function (10, 26, 27, 66), in general they are more potent and efficacious cannabinoid receptor agonists than THC (11, 67). In addition, SC drugs have additional ingredients such as preservatives, additives, fatty acids, amides, esters, benzodiazepines, and O-desmethyltramadol- an active metabolite of the opioid medication tramadol (26, 31, 68). It is suggested that these additional compounds are probably added to these drugs in purposely to induce greater psychoactive effect, act as masking agents to confuse the identification of the main psychoactive substances within these drugs (31, 68).

Since 2008, at least 200 different types of SCs have been isolated from herbal mixtures in several countries (27, 69, 70). This wide variability of psychoactive compounds is probably a result of (a) the impermanent production processes of these drugs, and (b) again their ever-changing compositions in an attempt to dodge prevention and legal actions (31).

One of the earliest compounds that was identified as a psychoactive component in SC drugs is JWH-018. Contrary to THC, JWH-018 has four times the affinity for CB_1_ receptors and 10 times the affinity for the CB_2_ receptors (67). Later-on, additional cannabinomimetic compounds such as CP 47,497, cannabicyclohexanol, HU-210, and the fatty acid, oleamide have been detected in samples of SC drugs in different areas around the globe (10, 11). Prior studies examined and indicated the therapeutic potential of SCs; HU-210 proposed to have anti-depressant effects (71), HU-211 proposed to have anti-inflammatory effect on brain trauma (72), and nabilone has antiemetic and analgesic effects (73, 74). However, while these studies report an effect that is induced by exclusive compound, within SC based-drugs, a mixture these cannabinoids as well as additional psychoactive ingredients generates a condition that incudes synergistic interactions which may underline their extrema and unpredicted adverse effects (10, 31).

## Adverse health effects of cannabinoids

### Acute effects of cannabinoids

The intoxication effects of cannabis are characterized by cannabis users as; mild euphoria, relaxation, and a general pleasant feeling (1, 7). These desired psychotropic effects are considered to be related to the presence of THC (8, 9). In laboratory settings, cannabis and THC induce dose-related impairment in several functions including; motor coordination and executive function (38, 75–78).

Further undesired symptoms including anxiety, panic attacks, and psychotic episodes were associated with cannabis intoxication, all of which are most often reported by naïve users and vulnerable individuals (56, 79). Similar to cannabis, the intoxication of SCs may induce reactions such as relaxation, euphoria, perceptual alteration, altered sense of time, and mild cognitive impairments (80).

These cognitive alterations increase the risk of road accidents if cannabis or SC users drive while intoxicated (1, 15). Epidemiological evidence demonstrates that cannabis is the most common illicit drug to be detected in drivers involved in fatal road accidents or stopped for dangerous driving (1). Accordingly, cannabis use by drivers is associated with an increased risk of being involved in motor vehicle crashes (81). There is less epidemiological data on the association between SC drugs and road accidents, yet, several case studies have documented driving under influence of SCs. Musshoff et al. reported 8 drivers in Germany that were stopped by police for reckless driving. Several types of SCs were identified in all suspected serum samples, drivers showed slow responses, retarded movements and an impairment of fine motor skills (52). Yeakel and Logan described 12 cases of driving under the influence of SCs, in all cases drivers presented a generally poor motor coordination (53). In a Norwegian study conducted during a 7-week period, about 2.2% of 726 blood samples that were collected from drivers that were suspected to drive under the influence of drugs, confirmed positive for SCs and majority of the drivers were involved in vehicle accidents (54). Taken together it seems that as with driving under the influence of cannabis (1, 81), driving after SCs may also increase the risk of being involved in road accidents (10, 15).

Despite the resemblances, there are major differences between the effect of cannabis and SC drugs, both in terms of spectrum and intensity of these effects (13). Case report studies indicate a wide range of undesired somatic effects ranging in intensity from nausea to more severe symptoms such as psychomotor agitation, diaphoresis, and palpitations (13, 29). Some symptoms such as seizures; agitation, hypertension, emesis, and hypokalemia are features of SC intoxication and are not present even after consuming high doses of organic cannabis (29, 31, 33).

Psychotropic effects of SC intoxication vary significantly. Some users report a feeling of sedation while others experience agitation, fatigue, and flushes (29). This variation may occur due to different concentrations of SCs within different brands (27). Compared with the intoxication of organic cannabis products which have a slow effect and gradually fade (7, 82), SCs have a shorter duration and peak earlier (83). For example, the effects of JWH-018 last approximately for 1–2 h, while CP-47,497 induce effects for ~5–6 h (20). Moreover, some adverse effects such as anxiety, hallucinations, insomnia, and psychotic episodes may be experienced for days and weeks after consuming SC products (84).

### Chronic effects of cannabinoids

Several studies demonstrate an association between repeated cannabis use and long-lasting cognitive impairments (2, 39, 85, 86), and an increase in risk for developing a variety of mental disorders. These include anxiety (34, 35), bipolar disorder (36), depression (37), and schizophrenia (2, 25, 32). There is growing evidence that SC drugs are associated with severe negative psychiatric and medical conditions (10, 15, 29, 31). This evidence demonstrates that repeated exposures to SCs induce overall negative side-effects which are more severe and long-lasting than those related with THC (10, 15, 29, 31).

A recent study by Cohen et al. shows deficiencies in a variety of high-order cognitive functions observed among SC users compared with recreational cannabis users including working memory, attention and recall (18). Another study indicates that SC users who had acute psychotic disorder induced by SC drugs, show cognitive impairments similar to those of schizophrenic patients (87). These results are compatible with evidence from rodent studies that show that repeated exposures to SCs induces long-lasting behavioral and cognitive impairments that resemble rodent models of schizophrenia (88, 89).

There is evidence indicating adverse effects of cannabis on cardiovascular function (1, 44, 46). Studies demonstrate that cannabis use can increase the risk of serious cardiovascular condition, including artery thrombosis, vasospasm, and myocardial infarction (90). Cannabinoid agonists, as well as THC, increase heart rate in a dose-dependent manner (1). Therefore, it is likely that these effects are mediated through cannabinoid-agonist's increase in catecholamines and increased cardiac workload together with a decreased supply of oxygen (91). Since SC drugs contain psychoactive compounds which have much higher affinity at CB_1_ receptors (67), it is not surprising that there are several reports that indicate an association between SC use and serious cardiovascular problems such as myocardial infarction and tachycardia in both adults and adolescents (92, 93).

### Cannabinoids and psychosis

The association between cannabinoids and psychosis is reported and it is well-recognized (2, 77, 79, 94), yet, casual relations between these two factors were not established (2, 79). However, converging evidence suggests that cannabis use has the potential for inducing psychosis (31, 77, 79, 95–98). SC products contain compounds which act as highly potent CB_1_ and CB_2_ full agonists, and in contrast to natural cannabis, contain no CBD (27, 31, 79). Due to the psychoactive features of SC drug ingredients it is not surprising that there are numerous reports on healthy and vulnerable individuals who suffer from recurrent psychosis after an acute or repeated consumption of SC drugs (15, 45, 99). Recent reports in Europe suggest that 15% of SC users who report to emergency departments present psychotic symptoms (100). These figures are far greater compared to those using other types of psychoactive substance (100). In addition, compared with cannabis, psychotic symptoms that are associated with SC are more severe and gross, in some cases they can even last for weeks following last use (10, 99).

### Effects of cannabinoids on the central nervous system

A large number of recent studies present a range of functional and structural neuronal abnormalities associated with regular use of cannabis (32, 49, 50). Generally, cannabis users show volumetric, gray matter, and white matter structural changes in the brain, particular within limbic and prefrontal areas (49, 50, 101). In addition, pharmacological studies draw a link between cannabis use and alterations of dopamine synthesis (102). Greater dose of THC, and an earlier age of onset area associated with these neuronal alterations (2, 32).

Compared with cannabis, there are fewer brain imaging studies exploring the neural correlates of SC use. Nurmedov et al. compared 20 males who used SC products with 20 healthy control participants and reported that SC users showed smaller gray-matter volume in the thalamus and the cerebellum (47). Recently, Zorlu et al. found a reduction of white matter volumes in several brain regions including the left temporal lobe and subcortical structures among SC users (48). In another single case study, a young SC user reported severe symptoms induced following a voluntary abstinence from SC drugs. In this patient, dopamine D_2_ and D_3_ receptors availability was lower in the striatum and in extra-striatal regions in comparison with healthy control participants during abstinence but it recovered after treatment (103). These initial studies highlight some the neurotoxic potential of SC products but since they are still preliminary. Further research is warranted.

### Health hazards and withdrawal

In contrast to cannabis, the use of SC has been associated with severe hazardous health effects on multiple systems and with death (28, 104, 105). During the last 8 years in the United Kingdom, there were 510 reports associated with SC use that required urgent medical intervention (57). In USA there were 37,500 reported cases of seizures and 3,682 reported cases of poisonings related to SC use during 2014 (106). In addition, while there is no available documentation on an overdose death as a result of cannabis use (1), there are numbers of reports indicating a fatal outcome following consumption of SC drugs. Tait et al. identified at least 26 cases of individuals who used SC products and exhibited side-effect complications that have led to their death. The major complications were cardiovascular events, respiratory depression, pulmonary complications and acute kidney injury (107). Prolonged consumption of SC is associated with serious withdrawal symptoms including; agitation, irritability, anxiety, and mood swings (43). Some of these symptoms are similar to cannabis, yet, the differences in presentation may reflect the presence of extraneous psychoactive compounds, including amphetamine-like stimulants, and the high affinity of these SCs (45, 43).

## Conclusions

Cannabinoid based drugs became increasingly popular despite the risks associated with their use (31, 45, 57). While the psychotropic effects associated with natural cannabis are related to the presence of THC (1), SC products contain a wide range of high-potent full agonists of the cannabinoid receptors that induce “THC-like” effects, but they are more severe and enduring (10, 15, 29, 31). While cannabis use usually induces psychotropic effects such as euphoria, relaxation, and a general pleasant feeling, it is associated with severe side-effects (1). The use of SC drugs is associated with more undesired effects including; agitation, irritability, confusion, hallucinations, delusions, psychosis, and death (28, 104, 105).

Chronic use of SC is associated with a greater risk for developing serious mental health disorder than cannabis or other psychoactive substances (100). Chronic use of cannabinoids is associated with structural and functional neuronal alterations (15, 32, 49), these alterations are moderated by the age of onset and the type of cannabinoids (32). This is crucial since the use of SC drugs is widely popular among young adults and teenager (10, 11). In addition, legal measures to control the use of SCs are reported as not effective enough to diminished the use of these drugs (26, 31, 106, 108), thus, further prevention programs should be conducted (31). Prevention programs to control SC drugs use and distribution may be based on information communication technology (ICT) tools and target young adults and students (109–111). Initial research regarding the efficiency of ICT-based prevention programs indicated on promising results in monitoring and control NPS use and distribution (109, 111). In conclusion, SC drugs show greater toxicity than organic cannabis, and therefore further investigation of their long-lasting and acute adverse effects is required as well as better detection and controlling measures against the spread of the use of SC products.

## Author contributions

All authors listed have made a substantial, direct and intellectual contribution to the work, and approved it for publication.

### Conflict of interest statement

The authors declare that the research was conducted in the absence of any commercial or financial relationships that could be construed as a potential conflict of interest.

## References

[1] Hall W Degenhardt L. Adverse health effects of non-medical cannabis use. Lancet (2009) 374:1383–91. 10.1016/S0140-6736(09)61037-0 19837255

[2] Volkow ND Swanson JM Evins AE DeLisi LE Meier MH Gonzalez R. Effects of cannabis use on human behavior, including cognition, motivation, and psychosis: a review. JAMA Psychiatry (2016) 73:292–7. 10.1001/jamapsychiatry.2015.3278 26842658

[3] UNDOC World Drug Report 2015. Vienna: United Nation Office on Drugs and Crime 2016 (2016).

[4] Borgelt LM Franson KL Nussbaum AM Wang GS. The pharmacologic and clinical effects of medical cannabis. Pharmacotherapy (2013) 33:195–209. 10.1002/phar.1187 23386598

[5] Amato L Minozzi S Mitrova Z Parmelli E Saulle R Cruciani F. Systematic review of safeness and therapeutic efficacy of cannabis in patients with multiple sclerosis, neuropathic pain, and in oncological patients treated with chemotherapy. Epidemiol Preven. (2017) 41:279–93. 10.19191/EP17.5-6.AD01.069 29119763

[6] Whiting PF Wolff RF Deshpande S Di Nisio M Duffy S Hernandez AV. Cannabinoids for medical use: a systematic review and meta-analysis. JAMA (2015) 313:2456–73. 10.1001/jama.2015.6358 26103030

[7] Ashton CH. Pharmacology and effects of cannabis: a brief review. Br J Psychiatry (2001) 178:101–6. 10.1192/bjp.178.2.101 11157422

[8] Gaoni Y Mechoulam R Isolation, structure, and partial synthesis of an active constituent of hashish. J Am Chem Soc. (1964) 86:1646–7. 10.1021/ja01062a046

[9] Pertwee RG Pharmacological actions of cannabinoids. In Pertwee R. G. editor. Handbook of Experimental Pharmacology, Vol. 168 New York, NY: Springer (2005). p. 1–51. 10.1007/3-540-26573-2_1 16596770

[10] Castaneto MS Gorelick DA Desrosiers NA Hartman RL Pirard S Huestis MA. Synthetic cannabinoids: epidemiology, pharmacodynamics, and clinical implications. Drug Alcohol Depend. (2014) 144:12–41. 10.1016/j.drugalcdep.2014.08.005 25220897 PMC4253059

[11] Gunderson EW Haughey HM Ait-Daoud N Joshi AS Hart CL. A survey of synthetic cannabinoid consumption by current cannabis users. Subst Abuse (2014) 35:184–9. 10.1080/08897077.2013.846288 24821356 PMC4048873

[12] Winstock A Lynskey M Borschmann R Waldron J. Risk of emergency medical treatment following consumption of cannabis or synthetic cannabinoids in a large global sample. J Psychopharmacol. (2015) 29:698–703. 10.1177/0269881115574493 25759401

[13] Spaderna M Addy PH D'Souza DC. Spicing things up: synthetic cannabinoids. Psychopharmacology (2013) 228:525–40. 10.1007/s00213-013-3188-4 23836028 PMC3799955

[14] Tournebize J Gibaja V Kahn JP. Acute effects of synthetic cannabinoids: update 2015. Subst Abuse (2017) 38:344–66. 10.1080/08897077.2016.1219438 27715709

[15] Weinstein AM Rosca P Fattore L London ED. Synthetic cathinone and cannabinoid designer drugs pose a major risk for public health. Front Psychiatry (2017) 8:156. 10.3389/fpsyt.2017.00156 28878698 PMC5572353

[16] Cohen K Weinstein A. The effects of cannabinoids on executive functions: evidence from cannabis and synthetic cannabinoids—a systematic review. Brain Sci. (2018) 8:40. 10.3390/brainsci8030040 29495540 PMC5870358

[17] Iseger TA Bossong MG. A systematic review of the antipsychotic properties of cannabidiol in humans. Schizophr Res. (2015) 162:153–61. 10.1016/j.schres.2015.01.033 25667194

[18] Cohen K Kapitány-Fövény M Mama Y Arieli M Rosca P Demetrovics Z. The effects of synthetic cannabinoids on executive function. Psychopharmacology (2017) 234:1121–34. 10.1007/s00213-017-4546-4 28160034

[19] Loeffler G Delaney E Hann M. International trends in spice use: prevalence, motivation for use, relationship to other substances, and perception of use and safety for synthetic cannabinoids. Brain Res Bull. (2016) 126:8–28. 10.1016/j.brainresbull.2016.04.013 27108542

[20] Winstock AR Barratt MJ. Synthetic cannabis: a comparison of patterns of use and effect profile with natural cannabis in a large global sample. Drug Alcohol Depend. (2013) 131:106–11. 10.1016/j.drugalcdep.2012.12.011 23291209

[21] Weaver MF Hopper JA Gunderson EW. Designer drugs 2015: assessment and management. Addict Sci Clin Pract. (2015) 10:10–19. 10.1186/s13722-015-0024-7 25928069 PMC4422150

[22] Egan KL Suerken CK Reboussin BA Spangler J Wagoner KG Sutfin EL. K2 and Spice use among a cohort of college students in southeast region of the USA. Am J Drug Alcohol Abuse (2015) 41:317–22. 10.3109/00952990.2015.1043438 26030768 PMC4526379

[23] Palamar JJ Acosta P. Synthetic cannabinoid use in a nationally representative sample of US high school seniors. Drug Alcohol Depend. (2015) 149:194–202. 10.1016/j.drugalcdep.2015.01.044 25736618 PMC4361370

[24] Cottencin O Rolland B Karila L. New designer drugs (synthetic cannabinoids and synthetic cathinones): review of literature. Curr Pharm Design (2013) 20:4106–11. 10.2174/13816128113199990622 24001292

[25] Di Forti M Marconi A Carra E Fraietta S Trotta A Bonomo M. Proportion of patients in south London with first-episode psychosis attributable to use of high potency cannabis: a case-control study. Lancet Psychiatry (2015) 2:233–8. 10.1016/S2215-0366(14)00117-5 26359901

[26] Fattore L Fratta W. Beyond THC: the new generation of cannabinoid designer drugs. Front Behav Neurosci. (2011) 5:60. 10.3389/fnbeh.2011.00060 22007163 PMC3187647

[27] van Amsterdam J Brunt T van den Brink W. The adverse health effects of synthetic cannabinoids with emphasis on psychosis-like effects. J Psychopharmacol. (2015) 29:254–63. 10.1177/0269881114565142 25586398

[28] Hermanns-Clausen M Kneisel S Szabo B Auwärter V. Acute toxicity due to the confirmed consumption of synthetic cannabinoids: clinical and laboratory findings. Addiction (2013) 108:534–44. 10.1111/j.1360-0443.2012.04078.x 22971158

[29] Seely KA Lapoint J Moran JH Fattore L. Spice drugs are more than harmless herbal blends: a review of the pharmacology and toxicology of synthetic cannabinoids. Progr NeuroPsychopharmacol Biol Psychiatry (2012) 39:234–43. 10.1016/j.pnpbp.2012.04.017 22561602 PMC3936256

[30] Morrison PD Zois V McKeown DA Lee TD Holt DW Powell JF. The acute effects of synthetic intravenous Δ9-tetrahydrocannabinol on psychosis, mood and cognitive functioning. Psychol Med. (2009) 39:1607–16. 10.1017/S0033291709005522 19335936

[31] Fattore L. Synthetic cannabinoids—further evidence supporting the relationship between cannabinoids and psychosis. Biol Psychiatry (2016) 79:539–48. 10.1016/j.biopsych.2016.02.001 26970364

[32] Lorenzetti V Solowij N Yücel M The role of cannabinoids on neuroanatomical alterations in cannabis users. Biol Psychiatry (2015) 79:17–31. 10.1016/j.biopsych.2015.11.013 26858212

[33] Castellanos D Singh S Thornton G Avila M Moreno A. Synthetic cannabinoid use: a case series of adolescents. J Adolesc Health (2011) 49:347–9. 10.1016/j.jadohealth.2011.08.002 21939863

[34] Crippa JA Zuardi AW Martín-Santos R Bhattacharyya S Atakan Z McGuire P. Cannabis and anxiety: a critical review of the evidence. Hum Psychopharmacol. (2009) 24:515–23. 10.1002/hup.1048 19693792

[35] Hill KP Cannabis use and risk for substance use disorders and mood or anxiety disorders. JAMA (2017) 317:1070–1. 10.1001/jama.2016.19706 28291877

[36] Weinstock LM Gaudiano BA Wenze SJ Epstein-Lubow G Miller IW. Demographic and clinical characteristics associated with comorbid cannabis use disorders (CUDs) in hospitalized patients with bipolar I disorder. Comprehens Psychiatry (2016) 65:57–62. 10.1016/j.comppsych.2015.10.003 26773991 PMC4715863

[37] Degenhardt L Hall W Lynskey M. Exploring the association between cannabis use and depression. Addiction (2003) 98:1493–504. 10.1046/j.1360-0443.2003.00437.x 14616175

[38] Curran VH Brignell C Fletcher S Middleton P Henry J Cognitive and subjective dose-response effects of acute oral Δ9-tetrahydrocannabinol (THC) in infrequent cannabis users. Psychopharmacology (2002) 164:61–70. 10.1007/s00213-002-1169-0 12373420

[39] Broyd SJ van Hell HH Beale C Yücel M Solowij N. Acute and chronic effects of cannabinoids on human cognition—a systematic review. Biol Psychiatry (2016) 79:557–67. 10.1016/j.biopsych.2015.12.002 26858214

[40] Weinstein A Brickner O Lerman H Greemland M Bloch M Lester H. Brain imaging study of the acute effects of Δ9-tetrahydrocannabinol (THC) on attention and motor coordination in regular users of marijuana. Psychopharmacology (2008) 196:119–31. 10.1007/s00213-007-0940-7 17899017

[41] Crean RD Crane NA Mason BJ. An evidence based review of acute and long-term effects of cannabis use on executive cognitive functions. J Addict Med. (2011) 5:1–8. 10.1097/ADM.0b013e31820c23fa 21321675 PMC3037578

[42] Pope HG Gruber AJ Hudson JI Huestis MA Yurgelun-Todd D. Neuropsychological performance in long-term cannabis users. Arch Gen Psychiatry (2001) 58:909–15. 10.1001/archpsyc.58.10.909 11576028

[43] Nacca N Vatti D Sullivan R Sud P Su M Marraffa J. The synthetic cannabinoid withdrawal syndrome. J Addict Med. (2013) 7:296–8. 10.1097/ADM.0b013e31828e1881 23609214

[44] Pacher P Steffens S Haskó G Schindler TH Kunos G. Cardiovascular effects of marijuana and synthetic cannabinoids: the good, the bad, and the ugly. Nat Rev Cardiol. (2018) 15:151. 10.1038/nrcardio.2017.130 28905873

[45] Davidson C Opacka-Juffry J Arevalo-Martin A Garcia-Ovejero D Molina-Holgado E Molina-Holgado F Spicing up pharmacology: a review of synthetic cannabinoids from structure to adverse events. In: Kendall D Alexander SPH editors. Advances in Pharmacology, Vol. 80, San Diego, CA: Elsevier (2017). p. 135–68. 10.1016/bs.apha.2017.05.001 28826533

[46] Reece AS Norman A Hulse GK. Cannabis exposure as an interactive cardiovascular risk factor and accelerant of organismal ageing: a longitudinal study. BMJ Open (2016) 6:91–118. 10.1136/bmjopen-2016-011891 27821595 PMC5129004

[47] Nurmedov S Metin B Ekmen S Noyan O Yilmaz O Darcin A. Thalamic and cerebellar gray matter volume reduction in synthetic cannabinoids users. Eur Addict Res. (2015) 21:315–20. 10.1159/000430437 26021304

[48] Zorlu N Di Biase MA Kalayci ÇÇ Zalesky A Bagci B Oguz N. Abnormal white matter integrity in synthetic cannabinoid users. Eur Neuropsychopharmacol. (2016) 26:1818–25. 10.1016/j.euroneuro.2016.08.015 27617779

[49] Batalla A Bhattacharyya S Yücel M Fusar-Poli P Crippa JA Nogué S. Structural and functional imaging studies in chronic cannabis users: a systematic review of adolescent and adult findings. PLoS ONE (2013) 8:e55821. 10.1371/journal.pone.0055821 23390554 PMC3563634

[50] Weinstein A Livny A Weizman A. Brain imaging studies on the cognitive, pharmacological and neurobiological effects of cannabis in humans: evidence from studies of adult users. Curr Pharm Des. (2016) 22:6366–79. 10.2174/1381612822666160822151323 27549374

[51] Hayatbakhsh MR O'Callaghan MJ Mamun AA Williams GM Clavarino A Najman JM. Cannabis use and obesity and young adults. Am J Drug Alcohol Abuse (2010) 36:350–6. 10.3109/00952990.2010.500438 20936991

[52] Musshoff F Madea B Kernbach-Wighton G Bicker W Kneisel S Hutter M. Driving under the influence of synthetic cannabinoids (“Spice”): a case series. Int J Legal Med. (2014) 128:59–64. 10.1007/s00414-013-0864-1 23636569

[53] Yeakel JK Logan BK. Blood synthetic cannabinoid concentrations in cases of suspected impaired driving. J Analyt Toxicol. (2013) 37:547–51. 10.1093/jat/bkt065 23965292

[54] Tuv SS Krabseth H Karinen R Olsen KM Øiestad EL Vindenes V. Prevalence of synthetic cannabinoids in blood samples from Norwegian drivers suspected of impaired driving during a seven weeks period. Accid Anal Prev. (2014) 62:26–31. 10.1016/j.aap.2013.09.009 24129318

[55] Tetrault JM Crothers K Moore BA Mehra R Concato J Fiellin DA. Effects of marijuana smoking on pulmonary function and respiratory complications: a systematic review. Arch Intern Med. (2007) 167:221–8. 10.1001/archinte.167.3.221 17296876 PMC2720277

[56] Hall W Solowij N. Adverse effects of cannabis. Lancet (1998) 352:1611–6. 10.1016/S0140-6736(98)05021-1 9843121

[57] Waugh J Najafi J Hawkins L Hill SL Eddleston M Vale JA. Epidemiology and clinical features of toxicity following recreational use of synthetic cannabinoid receptor agonists: a report from the United Kingdom National Poisons Information Service. Clin Toxicol. (2016) 54:512–8. 10.3109/15563650.2016.1171329 27091041

[58] Loeffler G Hurst D Penn A Yung K Spice, bath salts, and the US military: the emergence of synthetic cannabinoid receptor agonists and cathinones in the US Armed Forces. Mil Med. (2012) 177:1041–8. 10.7205/MILMED-D-12-00180 23025133

[59] Brewer TL Collins M. A review of clinical manifestations in adolescent and young adults after use of synthetic cannabinoids. J Spec Pediatr Nurs. (2014) 19:119–26. 10.1111/jspn.12057 24320158

[60] Huffman JW Bushell SM Miller JRA Wiley JL Martin BR 1-Methoxy-, 1-deoxy-11-hydroxy-and 11-hydroxy-1-methoxy-Δ 8-tetrahydrocannabinols: new selective ligands for the CB 2 receptor. Bioorg Med Chem. (2002) 10:4119–29. 10.1016/S0968-0896(02)00331-0 12413866

[61] Rhee MH Vogel Z Barg J Bayewitch M Levy R Hanuš L. Cannabinol derivatives: binding to cannabinoid receptors and inhibition of adenylylcyclase. J Med Chem. (1997) 40:3228–33. 10.1021/jm970126f 9379442

[62] Mechoulam R Peters M Murillo-Rodriguez E Hanus LO. Cannabidiol–recent advances. Chem Biodivers. (2007) 4:1678–92. 10.1002/cbdv.200790147 17712814

[63] Leweke FM Schneider U Radwan M Schmidt E Emrich HM. Different effects of nabilone and cannabidiol on binocular depth inversion in man. Pharmacol Biochem Behav. (2000) 66:175–81. 10.1016/S0091-3057(00)00201-X 10837858

[64] Englund A Morrison PD Nottage J Hague D Kane F Bonaccorso S. Cannabidiol inhibits THC-elicited paranoid symptoms and hippocampal-dependent memory impairment. J Psychopharmacol. (2013) 27:19–27. 10.1177/0269881112460109 23042808

[65] Bhattacharyya S Crippa JA Allen P Martin-Santos R Borgwardt S Fusar-Poli P. Induction of psychosis byδ9-tetrahydrocannabinol reflects modulation of prefrontal and striatal function during attentional salience processing. Arch Gen Psychiatry (2012) 69:27–36. 10.1001/archgenpsychiatry.2011.161 22213786

[66] Wiley JL Marusich JA Huffman JW. Moving around the molecule: relationship between chemical structure and *in vivo* activity of synthetic cannabinoids. Life Sci. (2014) 97:55–63. 10.1016/j.lfs.2013.09.011 24071522 PMC3944940

[67] Pertwee RG. Ligands that target cannabinoid receptors in the brain: from THC to anandamide and beyond. Addict Biol. (2008) 13:147–59. 10.1111/j.1369-1600.2008.00108.x 18482430

[68] Zuba D Byrska B. Prevalence and co-existence of active components of “legal highs.” Drug Test Anal. (2013) 5:420–9. 10.1002/dta.1365 22549997

[69] Hudson S Ramsey J. The emergence and analysis of synthetic cannabinoids. Drug Test Anal. (2011) 3:466–78. 10.1002/dta.268 21337724

[70] Uchiyama N Kikura-Hanajiri R Ogata J Goda Y. Chemical analysis of synthetic cannabinoids as designer drugs in herbal products. Forens Sci Int. (2010) 198:31–8. 10.1016/j.forsciint.2010.01.004 20117892

[71] Hill MN Gorzalka BB. Pharmacological enhancement of cannabinoid CB1 receptor activity elicits an antidepressant-like response in the rat forced swim test. Eur Neuropsychopharmacol. (2005) 15:593–99. 10.1016/j.euroneuro.2005.03.003 15916883

[72] Shohami E Novikov M Bass R. Long-term effect of HU-211, a novel non-competitive NMDA antagonist, on motor and memory functions after closed head injury in the rat. Brain Res. (1995) 674:55–62. 10.1016/0006-8993(94)01433-I 7773695

[73] Lewis IH Campbell DN Barrowcliffe MP. Effect of nabilone on nausea and vomiting after total abdominal hysterectomy. Br J Anaesth. (1994) 73:244–6. 10.1093/bja/73.2.244 7917745

[74] John Redmond W Goffaux P Potvin S Marchand S Analgesic and antihyperalgesic effects of nabilone on experimental heat pain. Curr Med Res Opin. (2008) 24:1017–24. 10.1185/030079908X280635 18302810

[75] Asmaro D Carolan PL Liotti M. Electrophysiological evidence of early attentional bias to drug-related pictures in chronic cannabis users. Addict Behav. (2014) 39:114–21. 10.1016/j.addbeh.2013.09.012 24126204

[76] Bolla KI Eldreth DA Matochik JA Cadet JL. Neural substrates of faulty decision-making in abstinent marijuana users. Neuroimage (2005) 26:480–92. 10.1016/j.neuroimage.2005.02.012 15907305

[77] D'Souza DC Perry E MacDougall L Ammerman Y Cooper T Wu Y. The psychotomimetic effects of intravenous delta-9-tetrahydrocannabinol in healthy individuals: implications for psychosis. Neuropsychopharmacology (2004) 29:1558–72. 10.1038/sj.npp.1300496 15173844

[78] Gorelick DA Goodwin RS Schwilke E Schwope DM Darwin WD Kelly DL. Tolerance to effects of high-dose oral Δ9-tetrahydrocannabinol and plasma cannabinoid concentrations in male daily cannabis smokers. J Analyt Toxicol. (2013) 37:11–6. 10.1093/jat/bks081 23074216 PMC3584989

[79] Burns JK. Pathways from cannabis to psychosis: a review of the evidence. Front Psychiatry (2012) 4:128. 10.3389/fpsyt.2013.00128 24133460 PMC3796266

[80] Wilson B Tavakoli H DeCecchis D Mahadev V Synthetic cannabinoids, synthetic cathinones, and other emerging drugs of abuse. Psychiatr Ann. (2013) 43:558–64. 10.3928/00485713-20131206-08

[81] Li MC Brady JE DiMaggio CJ Lusardi AR Tzong KY Li G. Marijuana use and motor vehicle crashes. Epidemiol Rev. (2011) 34:65–72. 10.1093/epirev/mxr017 21976636 PMC3276316

[82] Ramaekers JG Kauert G Theunissen EL Toennes SW Moeller MR. Neurocognitive performance during acute THC intoxication in heavy and occasional cannabis users. J Psychopharmacol. (2009) 23:266–77. 10.1177/0269881108092393 18719045

[83] Fantegrossi WE Moran JH Radominska-Pandya A Prather PL. Distinct pharmacology and metabolism of K2 synthetic cannabinoids compared to Δ 9-THC: mechanism underlying greater toxicity? Life Sci. (2014) 97:45–54. 10.1016/j.lfs.2013.09.017 24084047 PMC3945037

[84] Papanti D Schifano F Botteon G Bertossi F Mannix J Vidoni D. “Spiceophrenia”: a systematic overview of “Spice”-related psychopathological issues and a case report. Hum Psychopharmacol. (2013) 28:379–89. 10.1002/hup.2312 23881886

[85] Bossong M Jager G Bhattacharyya S Allen P. Acute and non-acute effects of cannabis on human memory function: a critical review of neuroimaging studies. Curr Pharm Des. (2014) 20:2114–25 10.2174/13816128113199990436. 23829369

[86] Solowij N Battisti R. The chronic effects of cannabis on memory in humans: a review. Curr Drug Abuse Rev. (2008) 1:81–98. 10.2174/1874473710801010081 19630708

[87] Altintas M Inanc L Oruc GA Arpacioglu S Gulec H. Clinical characteristics of synthetic cannabinoid-induced psychosis in relation to schizophrenia: a single-center cross-sectional analysis of concurrently hospitalized patients. Neuropsychiatr Dis Treat. (2016) 12:1893. 10.2147/NDT.S107622 27536110 PMC4977070

[88] Bambico FR Nguyen NT Katz N Gobbi G Chronic exposure to cannabinoids during adolescence but not during adulthood impairs emotional behaviour and monoaminergic neurotransmission. Neurobiol Dis. (2010) 37:641–55. 10.1016/j.nbd.2009.11.020 19969082

[89] Renard J Krebs MO Jay TM Le Pen G. Long-term cognitive impairments induced by chronic cannabinoid exposure during adolescence in rats: a strain comparison. Psychopharmacology (2013) 225:781–90. 10.1007/s00213-012-2865-z 22983145

[90] Jouanjus E Raymond V Lapeyre-Mestre M Wolff V. What is the current knowledge about the cardiovascular risk for users of cannabis-based products? A systematic review. Curr Atheroscl Rep. (2017) 19:1–15. 10.1007/s11883-017-0663-0 28432636

[91] Aryana A Williams MA Marijuana as a trigger of cardiovascular events: speculation or scientific certainty? Int J Cardiol. (2007) 118:141–4. 10.1016/j.ijcard.2006.08.001 17005273

[92] Barceló B Pichini S López-Corominas V Gomila I Yates C Busardò FP. Acute intoxication caused by synthetic cannabinoids 5F-ADB and MMB-2201: a case series. Forensic Sci Int. (2017) 273:10–14. 10.1016/j.forsciint.2017.01.020 28190538

[93] McIlroy G Ford L Khan JM. Acute myocardial infarction, associated with the use of a synthetic adamantyl-cannabinoid: a case report. BMC Pharmacol Toxicol. (2016) 17:1–4. 10.1186/s40360-016-0045-1 26772803 PMC4715335

[94] Radhakrishnan R Wilkinson ST D'Souza DC Gone to pot-a review of the association between cannabis and psychosis. Front Psychiatry (2014) 5:50–4. 10.3389/fpsyt.2014.00054 24904437 PMC4033190

[95] Bioque M Cabrera B García-Bueno B Mac-Dowell KS Torrent C Saiz PA. Dysregulated peripheral endocannabinoid system signaling is associated with cognitive deficits in first-episode psychosis. J Psychiatr Res. (2016) 75:14–21. 10.1016/j.jpsychires.2016.01.002 26783729

[96] Bloomfield MAP Morgan CJA Egerton A Kapur S Curran HV Howes OD. Dopaminergic function in cannabis users and its relationship to cannabis-induced psychotic symptoms. Biol Psychiatry (2014) 75:470–8. 10.1016/j.biopsych.2013.05.027 23820822

[97] D'Souza DC Sewell RA Ranganathan M. Cannabis and psychosis/schizophrenia: human studies. Eur Arch Psychiatry Clin Neurosci. (2009) 259:413–31. 10.1007/s00406-009-0024-2 19609589 PMC2864503

[98] Fergusson DM Poulton R Smith PF Boden JM. Cannabis and psychosis. BMJ (2006) 332:172–5. 10.1136/bmj.332.7534.172 16424500 PMC1336774

[99] Khan M Pace L Truong A Gordon M Moukaddam N. Catatonia secondary to synthetic cannabinoid use in two patients with no previous psychosis. Am J Addict. (2016) 25:25–7. 10.1111/ajad.12318 26781357

[100] Vallersnes OM Dines AM Wood DM Yates C Heyerdahl F Hovda KE Psychosis associated with acute recreational drug toxicity: a European case series. BMC Psychiatry (2016) 16:293 10.1186/s12888-016-1002-7 27538886 PMC4990880

[101] Yücel M Solowij N Respondek C Whittle S Fornito A Pantelis C. Regional brain abnormalities associated with long-term heavy cannabis use. Arch Gen Psychiatry (2008) 65:694–701. 10.1001/archpsyc.65.6.694 18519827

[102] Bloomfield MAP Ashok AH Volkow ND Howes OD. The effects of Δ9-tetrahydrocannabinol on the dopamine system. Nature (2016) 539:369–77. 10.1038/nature20153 27853201 PMC5123717

[103] Rominger A Cumming P Xiong G Koller G Förster S Zwergal A Effects of acute detoxification of the herbal blend “Spice Gold”on dopamine D 2/3 receptor availability: a [18 F] fallypride PET study. Eur Neuropsychopharmacol. (2013) 23:1606–10. 10.1016/j.euroneuro.2013.01.009 23452563

[104] Mir A Obafemi A Young A Kane C. Myocardial infarction associated with use of the synthetic cannabinoid K2. Pediatrics (2011) 128:e1622–7. 10.1542/peds.2010-3823 22065271

[105] Schaefer N Peters B Bregel D Kneisel S Auwärter V Schmidt PH A fatal case involving several synthetic cannabinoids. Toxichem Krimtech (2013) 80:248–51.

[106] Keyes KM Rutherford C Hamilton A Palamar JJ. Age, period, and cohort effects in synthetic cannabinoid use among US adolescents, 2011–2015. Drug Alcohol Depend. (2016) 166:159–67. 10.1016/j.drugalcdep.2016.07.018 27491817 PMC4996475

[107] Tait RJ Caldicott D Mountain D Hill SL Lenton S. A systematic review of adverse events arising from the use of synthetic cannabinoids and their associated treatment. Clin Toxicol. (2016) 54:1–13. 10.3109/15563650.2015.1110590 26567470

[108] Bilgrei OR From “herbal highs” to the “heroin of cannabis”: exploring the evolving discourse on synthetic cannabinoid use in a Norwegian Internet drug forum. Int J Drug Policy (2016) 29:1–8. 10.1016/j.drugpo.2016.01.011 26860324

[109] Corazza O Schifano F Simonato P Fergus S Assi S Stair J. Phenomenon of new drugs on the Internet: the case of ketamine derivative methoxetamine. Hum Psychopharmacol. (2012) 27:145–9. 10.1002/hup.1242 22389078

[110] Corazza O Assi S Trincas G Simonato P Corkery J Deluca P Novel Drugs, Novel Solutions: exploring the potentials of web-assistance and multimedia approaches for the prevention of drug abuse. Italian J Addict. (2011) 1:25–30.

[111] Corazza O Simonato P Corkery J Trincas G Schifano F. “Legal highs”: safe and legal “heavens”? A study on the diffusion, knowledge and risk awareness of novel psychoactive drugs among students in the UK. Riv Psichiatria (2014) 49:89–94. 10.1708/1461.16147 24770577

